# Benefits of specialisation in the management of pancreatic cancer: results of a Scottish population-based study

**DOI:** 10.1038/sj.bjc.6601999

**Published:** 2004-06-29

**Authors:** R W Parks, V Bettschart, S Frame, D L Stockton, D H Brewster, O J Garden

**Affiliations:** 1 Department of Clinical and Surgical Sciences, The University of Edinburgh, Royal Infirmary, 51Little France Crescent, Edinburgh EH16 4SA, UK; 2 Information and Statistics Division (NHS Scotland), Trinity Park House, South Trinity Road, Edinburgh EH5 3SQ, UK

**Keywords:** pancreatic cancer, specialisation, survival, resection, hospital volume, consultant volume

## Abstract

Pancreatic cancer is associated with a very poor prognosis; however, in selected patients, resection may improve survival. Several recent reports have demonstrated that concentration of treatment activity for patients with pancreatic cancer has resulted in improved outcomes. The aim of this study was to ascertain if there was any evidence of benefit for specialised care of patients with pancreatic cancer in Scotland. Records of patients diagnosed with pancreatic cancer during the period 1993–1997 were identified. Three indicators of co-morbidity were calculated for each patient. Operative procedures were classified as resection, other surgery or biliary stent. Prior to analysis, consultants were assigned as specialist pancreatic surgeons, clinicians with an interest in pancreatic disease or nonspecialists. Data were analysed with regard to 30-day mortality and survival outcome. The final study population included 2794 patients. The 30-day mortality following resection was 8%, and hospital or consultant volume did not affect postoperative mortality. The 30-day mortality rate following palliative surgical operations was 20%, and consultants with higher case loads or with a specialist pancreatic practice had significantly fewer postoperative deaths (*P*=0.014 and 0.002, respectively). For patients undergoing potentially curative or palliative surgery, the adjusted hazard of death was higher in patients with advanced years, increased co-morbidity, metastatic disease, and was lower for those managed by a specialist (RHR 0.63, 95% CI 0.50–0.78) or by a clinician with an interest in pancreatic disease (RHR 0.63, 0.48–0.82). The risk of death 3 years after diagnosis of pancreatic cancer is higher among patients undergoing surgical intervention by nonspecialists. Specialisation and concentration of cancer care has major implications for the delivery of health services.

Every year in the UK, around 7000 people are diagnosed with pancreatic cancer (Office for National Statistics, 2002), and more than 6500 are recorded as dying from the disease (Quinn *et al*, 2001). In contrast to many other cancers, there has been little improvement in survival prospects in recent decades, and currently, less than 3% of patients remain alive 5 years after diagnosis (Coleman *et al*, 1999; Scottish Cancer Intelligence Unit, 2000). Although this dismal prognosis has led to a nihilistic perspective by some (Gudjonsson, 1995), others have reported 5-year survival rates following pancreatic resection in excess of 20% (Yeo *et al*, 1995). Furthermore, it has recently been suggested that there may be some scope for improvement in survival figures in the UK (NHS Executive, 2001; Garden, 2001). This guarded optimism is based on accumulating evidence that the concentration of treatment activity among fewer high volume hospitals and/or surgeons (as a proxy for specialisation) may lead to improved outcomes for patients with pancreatic cancer (Edge *et al*, 1993; Gordon *et al*, 1995; Lieberman *et al*, 1995; Neoptolemos *et al*, 1997; Gouma *et al*, 2000; NHS Executive, 2001; Garden, 2001; Rosemurgy *et al*, 2001; Teisberg *et al*, 2001; Halm *et al*, 2002; Birkmeyer *et al*, 2002; Bachmann *et al*, 2003). Many of the studies report reduced postoperative morbidity and mortality rates, and shorter postoperative hospital stays following resection of pancreatic cancer, although few have analysed long-term outcome. Recently, it has also been shown that increased hospital volume is associated with a decreased hospital mortality rate for palliative bypass procedures and for stent insertion as treatment for malignant obstructive jaundice (Sosa *et al*, 1998). These phenomena have been related to the importance of a multidisciplinary approach to both curative and palliative procedures available in high volume provider institutions for patients with pancreatic cancer.

The aim of the present study was to investigate whether there is any evidence of benefit from specialised care of patients with pancreatic cancer in Scotland, and by inference, whether there is any scope nationally to improve the outlook of this unfortunate group of patients.

## DATA AND METHODS

The records of residents of Scotland diagnosed with pancreatic cancer (ICD-9157; ICD-10 C25) during the 5-year period 1993–1997 were selected from the Scottish Cancer Registry (SCR). During this period, the age-standardised incidence rate (world standard population) per 100 000 was 7.2 in males and 5.3 in females (Parkin *et al*, 2002). Equivalent mortality rates for the same period were 6.7 in males and 4.9 in females (World Health Organization, 2001). SCR is a population-based registry that covers the whole of Scotland, with a catchment population of around 5.1 million. Data quality is believed to be high, both in terms of reliability (Brewster *et al*, 2002) and completeness of ascertainment (Brewster *et al*, 1997). However, in common with many other countries, a high proportion of pancreatic cancers are not microscopically verified (Parkin *et al*, 2002), precise subsite of origin is often not established or available, and stage of disease at diagnosis is not collected routinely. Excluded from further analysis were 178 cases registered on the basis of a death certificate only (DCO), and 67 nonacinar cell pancreatic cancers (neuroendocrine tumours (*n*=27), sarcoma (*n*=4), cholangiocarcinoma (*n*=8), and other tumour types rarely associated with pancreas as the primary site of origin (*n*=28)). Although mainly not microscopically verified, tumours coded as primary malignant neoplasms, not otherwise specified (NOS) were included on the basis that they were more likely than not to be carcinomas.

SCR records were supplemented with information on co-morbidity, metastatic disease and operative procedures from hospital discharge (SMR01) records, derived from a permanently linked database of hospital discharges (including day cases), cancer registrations, and deaths (Kendrick and Clarke, 1993). There is evidence that the coding of hospital discharge records in Scotland is reliable, at least for the main variables, such as primary operative procedure (Harley and Jones, 1996). Three indicators of co-morbidity were calculated for each member of the cohort: two of these – the Charlson score (Charlson *et al*, 1987) and the Scottish index (Clinical Outcomes Working Group, 1996) – were based on selected diagnoses found in any position on SMR01 records within 5 years of the incidence date on the index cancer registration record. The third co-morbidity index was based on the number of bed-days of hospitalisation in the 5 years prior to diagnosis of pancreatic cancer. To avoid confounding due to the often insidious onset of symptoms and signs of pancreatic cancer, this index was calculated for the period between 6 months and 5 years prior to diagnosis, and separately for the 6 months immediately prior to diagnosis.

Operative procedures are coded on SMR01 records according to OPCS4 (Office of Population Censuses and Surveys, 1990), a widely used UK classification of surgical operations and procedures. Based on these codes, inpatient and daycase operative procedures were classified as resection, other surgery (predominantly palliative bypass operations), or biliary stent. Prior to analysis, and based on local knowledge, consultants were assigned to three categories: specialist pancreatic surgeons, clinicians with an interest in the pancreas, or nonspecialists. Specialists were defined as those clinicians who had a recognised multidisciplinary team with an associated oncologist and pathologist, who contributed to clinical trials, and who had an established audit system and regularly reported their results locally and nationally. Clinicians with an interest in the pancreas were recognised as those who had a broader interest in upper gastrointestinal conditions but who were known for co-ordinating the assessment and management of patients with pancreatic cancer. Nonspecialists were defined as those who had a more general clinical practice or were recognised as having a specific interest in another surgical subspecialty.

Persons diagnosed with pancreatic cancer during 1993–1997 were assigned to a 1991 census-based Carstairs deprivation quintile according to their postcode sector of residence at the time of diagnosis (Morris and Carstairs, 1991). The Carstairs deprivation score is a small area indicator of socioeconomic status based on the prevalence measured at the decennial census of four characteristics: overcrowding, male unemployment, social class, and car ownership. Deprivation quintile one represents the least deprived areas of Scotland and deprivation quintile five the most deprived areas.

### Statistical methods

The *χ*^2^-test was used to assess the statistical significance of differences in 30-day postoperative mortality rates according to age-group, year of diagnosis, hospital caseload, consultant caseload, and consultant specialisation category. Kaplan–Meier survival analysis was used to obtain estimates of crude survival at 3 years following diagnosis. The log-rank test was used to test for equality of survival curves between patients treated by resection and all other patients. Multivariate Cox's proportional hazards models (Collett, 1994) were used to assess separately the impact of patient-, tumour- and health service-related factors on survival at 3 years following diagnosis. A final model was based on the statistically significant factors from the three separate models. The end point for these analyses was death from any cause.

## RESULTS

The final study population included 2794 patients with the characteristics shown in Table 1

**Table 1:** Characteristics of the study population

**Factor**	**No. of cases**	**%**
*Year of diagnosis*		
1993	553	19.8
1994	552	19.8
1995	479	17.1
1996	610	21.8
1997	600	21.5
		
*Age groups*		
0–39	14	0.5
40–49	93	3.3
50–59	312	11.2
60–69	717	25.7
70–79	946	33.9
⩾80	712	25.5
		
*Sex*		
Male	1330	47.6
Female	1464	52.4
		
*Carstairs deprivation quintile*		
(Least deprived) 1	510	18.3
2	600	21.5
3	594	21.3
4	527	18.9
(Most deprived) 5	562	20.1
Unknown	1	0.0
		
*Previous cancer*		
No	2611	93.5
Yes	183	6.6
		
*Charlson co-morbidity index*^a^		
None	2160	77.3
1–2 conditions	95	3.4
⩾3 conditions	539	19.3
		
*Scottish co-morbidity index*^a^		
None	1994	71.4
1 condition	516	18.5
⩾2 conditions	284	10.2
		
*Bed-days co-morbidity index (in 6 months prior to diagnosis)*^a^		
None	334	12
1–4 days	284	10.2
5–10 days	453	16.2
⩾11 days	1723	61.7
		
*Bed-days co-morbidity index (between 6 months and 5 years prior to diagnosis)*^a^		
None	1555	55.7
1–4 days	290	10.4
5–10 days	296	10.6
⩾11 days	653	23.4
		
*Tumour subsite*		
Head of pancreas	1263	45.2
Body of pancreas	112	4.0
Tail of pancreas	57	2.0
Other	40	1.4
Unspecified	1322	47.3
		
*Tumour morphology*		
Neoplasm, not otherwise specified	222	8.0
Carcinoma, not otherwise specified	1523	54.5
Adenocarcinoma, not otherwise specified	902	32.3
Mucin-producing adenocarcinoma	51	1.8
Other	96	3.4
		
*Microscopically verified*		
No	1616	57.8
Yes	1178	42.2
		
*Metastases within 4 weeks of diagnosis*^a^		
No	2023	72.4
Yes	771	27.6
		
*Presence of ascites recorded*^a^		
No	2674	95.7
Yes	120	4.3
		
*Surgical resection*^a^		
No	2663	95.3
Yes	131	4.7
		
*Other surgery*^a^		
No	2102	75.2
Yes	692	24.8
		
*Biliary stent*^a^		
No	2054	73.5
Yes	740	26.5
		
*Chemotherapy*^a^		
No	2646	94.7
Yes	148	5.3
		
*Radiotherapy*^a^		
No	2769	99.1
Yes	25	0.9
		
*Caseload of main treating consultant*^a^		
Unknown	29	1.0
<5 cases	799	28.6
5–9 cases	612	21.9
10–19 cases	460	16.5
⩾20 cases	894	32.0
		
*Caseload of hospital of treatment*^a^		
Unknown	29	1.0
1 case	43	1.6
2–9 cases	113	4.1
10–29 cases	132	4.8
⩾30 cases	2477	89.6
		
*Specialty of main treating consultant*^a^		
Specialist	230	8.2
Clinician with an interest in the pancreas	109	3.9
Nonspecialist	2426	86.8
Unknown	29	1.0
Total	2794	100

a Information derived from linked hospital discharge records does not include outpatient diagnostic or procedural information.

. Although the age-standardised incidence of pancreatic cancer is known to be higher in males than females, the absolute number of cases is higher in females, reflecting the age and sex structure of the Scottish population. Some of the characteristics shown are based on information derived from linked hospital discharge records, and do not include outpatient diagnostic or procedural information. Thus, for example, the number of patients receiving chemotherapy and radiotherapy may be underestimated.

Table 2

**Table 2:** 30-day postoperative mortality rates for patients undergoing surgery or biliary stenting by age group, year of diagnosis, hospital workload, workload of main treating consultant, and specialty of main treating consultant (*P*-values are shown for statistically significant differences)

	**Resection^a^**		**Other surgery^a^**		**Biliary stent^a^**	
**Factor**	**No. of cases**	**No. (%) of deaths**	**No. of cases**	**No. (%) of deaths**	**No. of cases**	**No. (%) of deaths**
*Age groups*			*P*=0.016		*P*=0.023	
0–39	1	0	6	0	5	0
40–49	14	2 (14%)	24	3 (13%)	19	2 (11%)
50–59	30	1 (3%)	109	9 (8%)	78	7 (9%)
60–69	63	4 (6%)	215	36 (17%)	202	22 (11%)
70–79	21	3 (14%)	239	57 (24%)	261	51 (20%)
⩾80	2	0	99	33 (33%)	175	45 (26%)
						
*Year of diagnosis*			*P*<0.001		*P*=0.001	
1993	21	2 (10%)	158	36 (23%)	119	21 (18%)
1994	23	3 (13%)	153	43 (28%)	116	31 (27%)
1995	24	3 (13%)	129	22 (17%)	148	29 (20%)
1996	32	1 (3%)	144	23 (16%)	183	25 (14%)
1997	31	1 (3%)	108	14 (13%)	174	21 (12%)
						
*Caseload of hospital of treatment*^a^						
1 case	0	N/A	0	N/A	0	N/A
2–9 cases	0	N/A	10	2 (20%)	1	0
10–29 cases	0	N/A	33	12 (36%)	18	4 (22%)
⩾30 cases	131	10 (8%)	649	124 (19%)	721	123 (17%)
						
*Caseload of main treating consultant*^a^			*P*=0.014			
1 case	7	1 (14%)	102	21 (21%)	88	18 (21%)
2–4 cases	11	3 (27%)	177	37 (21%)	112	24 (21%)
5–9 cases	18	1 (6%)	176	47 (27%)	108	16 (15%)
⩾10 cases	95	5 (5%)	237	33 (14%)	432	69 (16%)
						
*Specialty of main treating consultant*^a^			*P*=0.002			
Specialist	51	1 (2%)	86	6 (7%)	120	13 (11%)
Clinician with an interest in the pancreas	32	2 (6%)	50	7 (14%)	33	7 (21%)
Nonspecialist	48	7 (15%)	556	125 (23%)	587	107 (18%)
						
Total	131	10 (8%)	692	138 (20%)	740	127 (17%)

a Information derived from linked hospital discharge records does not include outpatient diagnostic or procedural information. Note that patients can appear in more than one of the treatment groups. N/A: not applicable.

shows the crude 30-day postoperative mortality rates for patients undergoing surgery or biliary stenting by age-group, year of diagnosis, hospital workload, workload of main treating consultant, and consultant specialisation category. Only 4.7% of the entire cohort of patients underwent potentially curative surgical resection and the relatively small number of events (10 deaths) mitigate against detecting statistically significant differences between different categories. The overall 30-day postoperative mortality following pancreatic resection was 8%, with a tendency to be lower in the two most recent years of diagnosis (3%), although this was not statistically significant. There was no obvious effect of hospital volume on postoperative mortality, although very few patients were treated in hospitals dealing with less than 10 cases. Postoperative mortality was generally lower among patients undergoing potentially curative resection when treated by consultants with higher caseloads or pancreatic specialists, but these differences did not achieve statistical significance (*P*=0.062 and 0.058, respectively).

The overall 30-day postprocedural mortality following palliative pancreatic surgery and biliary stenting was 20 and 17%, respectively. Mortality was significantly higher among the elderly, and significantly lower in recent years of diagnosis (Table 2). Significant differences in postoperative mortality rates were demonstrated in patients undergoing palliative surgical operations as consultants with higher case loads or with a specialist pancreatic practice had fewer postoperative deaths (*P*=0.014 and 0.002, respectively).

Crude survival at 3 years after diagnosis was 19% in those patients undergoing resection, but only 2% in other patients (*P*<0.001). Table 3

**Table 3:** Adjusted^a^ hazard ratios of death (and 95% confidence intervals) within 3 years of diagnosis

	**All patients**		**Surgical patients^b^**	
**Factor**	**HR (95% CI)**	***P*-value**	**HR (95% CI)**	***P***-**value**
*Year of diagnosis*			No statistically significant differences	
1993	1.00			
1994	0.98 (0.87, 1.11)	0.779		
1995	0.94 (0.83, 1.06)	0.309		
1996	0.81 (0.72, 0.92)	0.001		
1997	0.88 (0.78, 1.00)	0.043		
				
*Age groups*				
0–49	1.00		1.00	
50–59	0.95 (0.75, 1.19)	0.648	1.00 (0.69, 1.46)	0.982
60–69	1.14 (0.92, 1.41)	0.241	1.26 (0.88, 1.78)	0.205
70–79	1.14 (0.92, 1.41)	0.245	1.41 (0.99, 2.02)	0.06
⩾80	1.41 (1.12, 1.76)	0.003	1.83 (1.22, 2.75)	0.003
				
*Sex*			No statistically significant differences	
Male	1.00			
Female	0.92 (0.85, 1.00)	0.042		
				
*Scottish co-morbidity index*^b^				
None	1.00		1.00	
1 condition	1.24 (1.12, 1.37)	<0.001	1.09 (0.91, 1.32)	0.351
⩾2 conditions	1.25 (1.09, 1.42)	0.001	1.51 (1.12, 2.05)	0.007
				
*Bed-days co-morbidity index (in 6 months prior to diagnosis)*^b^			No statistically significant differences	
None	1.00			
1–4 days	1.11 (0.94, 1.30)	0.226		
5–10 days	1.20 (1.03, 1.39)	0.017		
⩾11 days	1.33 (1.17, 1.50)	<0.001		
				
*Tumour subsite*				
Head of pancreas	1.00		1.00	
Body of pancreas	1.01 (0.83, 1.24)	0.918	1.19 (0.78, 1.83)	0.425
Tail of pancreas	0.97 (0.74, 1.28)	0.849	1.18 (0.55, 2.54)	0.665
Other	1.12 (0.81, 1.55)	0.499	0.86 (0.48, 1.52)	0.595
Unspecified	1.20 (1.10, 1.30)	<0.001	1.34 (1.15, 1.56)	<0.001
				
*Tumour morphology*	No statistically significant differences			
Neoplasm, not otherwise specified			1.00	
Carcinoma, not otherwise specified			0.74 (0.51, 1.07)	0.107
Adenocarcinoma, not otherwise specified			0.62 (0.42, 0.93)	0.022
Mucin-producing adenocarcinoma			0.48 (0.27, 0.85)	0.011
Other			0.39 (0.23, 0.66)	<0.001
				
*Microscopically verified*	No statistically significant differences			
No			1.00	
Yes			0.68 (0.55, 0.85)	0.001
				
*Metastases within 4 weeks of diagnosis*^b^				
No	1.00		1.00	
Yes	1.92 (1.76, 2.10)	<0.001	1.93 (1.60, 2.33)	<0.001
				
*Surgical resection*^b^			Not applicable	
No	1.00			
Yes	0.38 (0.31, 0.46)	<0.001		
				
*Other surgery*^b^			Not applicable	
No	1.00			
Yes	0.74 (0.67, 0.81)	<0.001		
				
*Biliary stent*^b^				
No	1.00		1.00	
Yes	0.72 (0.65, 0.79)	<0.001	0.70 (0.57, 0.85)	<0.001
				
*Chemotherapy*^b^			No statistically significant differences	
No	1.00			
Yes	0.59 (0.50, 0.71)	<0.001		
				
*Caseload of main treating consultant*^b^			No statistically significant differences	
1 case	1.00			
2–4 cases	0.87 (0.78, 0.97)	0.013		
5–9 cases	0.90 (0.80, 1.02)	0.088		
⩾10 cases	0.85 (0.77, 0.95)	0.003		
				
*Specialty of main treating consultant*^b^	No statistically significant differences			
Nonspecialist			1.00	
Specialist			0.63 (0.50, 0.78)	<0.001
Clinician with an interest in the pancreas			0.63 (0.48, 0.82)	0.001

a For each model (all patients, surgical patients) the results for the factors shown are adjusted for all the other statistically significant factors in the table.

b Information derived from linked hospital discharge records does not include outpatient diagnostic or procedural information.

summarises the results of multivariate survival analysis, presented as adjusted hazard ratios, based on the final model derived from Cox's proportional hazard modelling. For the sake of clarity, only results that achieved (or approached) statistical significance are shown. For all patients, the adjusted hazard of death was higher in patients diagnosed in earlier years, older patients, males, patients with co-morbidity (based on two out of four indices), patients with unspecified subsite of tumour, patients with metastases, patients who did not undergo any form of surgical procedure or biliary stenting, patients who did not receive chemotherapy, and patients managed by consultants with a low caseload. For patients undergoing resection or another surgical procedure, the adjusted hazard of death was higher in older patients, patients with co-morbidity (based on the Scottish index), patients with unspecified subsite of tumour, patients with unspecified morphology, patients without microscopic verification of their disease, patients with metastases, patients who did not undergo biliary stenting, and patients who were not managed by a specialist pancreatic surgeon or a clinician with an interest in the field.

## DISCUSSION

The results of our study suggest that the risk of death by 3 years after diagnosis of pancreatic cancer is 37% lower among patients undergoing surgical intervention who are managed by specialist pancreatic surgeons or clinicians with an interest in this field. No advantage of specialisation was seen for the total patient population, but this probably reflects the fact that surgical resection offers the best chance of survival (Wade *et al*, 1994; Bramhall *et al*, 1995; Sener *et al*, 1999), at least for localised disease (Wade *et al*, 1995).

The resection rate of 4.7% in this study was low by international standards (Edge *et al*, 1993; Baumel *et al*, 1994; Nakao and Takagi, 1998), but may reflect the small number of patients managed by specialist pancreatic surgeons or clinicians with an interest in this field (12.1% of cohort). However, the resection rate did increase from 3.8% in 1993 to 5.2% in 1997. The development of regional cancer networks in the United Kingdom in recent years and the requirement for all patients with cancer to be discussed at multidisciplinary meetings may result in more patients being considered for potentially curative surgery. Bachmann *et al* (2003) suggested that patients referred to less specialised doctors or hospitals were less likely to be investigated thoroughly, or to undergo palliative or potentially curative treatment.

Several studies (Edge *et al*, 1993; Gordon *et al*, 1995; Lieberman *et al*, 1995; Neoptolemos *et al*, 1997; Gouma *et al*, 2000; Garden, 2001; NHS Executive, 2001; Rosemurgy *et al*, 2001; Teisberg *et al*, 2001; Birkmeyer *et al*, 2002; Halm *et al*, 2002; Bachmann *et al*, 2003) have suggested that specialisation in this field may yield better results in terms of postoperative mortality, surgical complications, and longer term survival, although other studies have not found this relationship (Wade *et al*, 1994, 1996), and the possibility of publication bias must be considered. Some previous studies are subject to the criticism that in-hospital mortality was used as an outcome, despite the fact that length of stay may be lower in patients treated by specialist pancreatic surgeons or hospitals with higher caseloads (Imperato *et al*, 1996; Sosa *et al*, 1998; Gordon *et al*, 1999; Simunovic *et al*, 1999; Rosemurgy *et al*, 2001). Most of the studies used hospital or surgeon caseload as a proxy for specialisation, and while this may be a reasonable assumption, others have argued that there is no proof that repeating a procedure hundreds of times necessarily guarantees competence (Loefler, 2000). In our own study, when all patients were considered together, survival was significantly higher for patients treated by consultants dealing with larger caseloads, but this factor was not significant in the model concerning only surgical patients. Although our categorisation of specialist status might be construed as subjective compared to any definition based on caseload, it was assigned before analysis.

A major strength of our study is the fact that it is population-based and not therefore subject to some of the potential biases inherent in single institution-based studies. However, the study is based on data collected routinely and not specifically to fulfil the aims of the study. Although data quality is believed to be reasonable (Harley and Jones, 1996; Brewster *et al*, 1997, 2002), it is unlikely to be as high nor as detailed as if the data had been collected prospectively. We were obliged to accept the reality that, in this and many other countries (Parkin *et al*, 2002), a high proportion of pancreatic cancers have no information on precise subsite of origin, and are not verified by microscopy. However, given the very poor survival prospects for patients registered with pancreatic cancer in Scotland (Scottish Cancer Intelligence Unit, 2000), it seems unlikely that the cancer registry includes many clinically diagnosed cases of pancreatic cancer that are actually cases of benign pancreatic disease. Restriction of our analyses to microscopically verified cases only would, in our opinion, have introduced a substantial risk of bias.

Given the importance of tumour stage as a prognostic variable (Wade *et al*, 1995), the absence of this variable is an acknowledged limitation, although its importance as a discriminating variable may be less among the cohort of patients undergoing surgery. Although we cannot exclude residual confounding as an explanation for our results, it seems counter-intuitive that surgical patients with more favourable subsites of origin, more limited disease, and requiring less technically demanding procedures would be referred selectively to specialist surgeons. In relation to the variables available for our study, it is likely that some misclassification exists, although if this is random with respect to specialist status, it will have attenuated rather than exaggerated the differences in outcome by this factor. The prognostic impact of other factors, such as age, presence of metastases at diagnosis and co-morbidity, is plausible and expected. Although microscopic verification of diagnosis appears to have no statistically significant effect on the survival of all patients combined, it is associated with higher survival in surgical patients, presumably because resection is always likely to result in tissue diagnosis.

It is interesting to note that, although almost 90% of patients were treated in high volume hospitals, slightly less than half were managed by consultants treating 10 or more cases (Table 1). Thus, any shift in referral patterns towards higher volume surgeons may not have a substantial impact on patient travel times.

When considering the policy implications of the present study and related studies, it is important to acknowledge that not everyone is convinced of the merits of specialisation which does have some theoretical disadvantages (Loefler, 2000). However, the body of evidence is now such that we would suggest that the onus is on sceptics to provide evidence that surgical specialisation offers no advantages, or at least that the disadvantages outweigh the benefits. From a pragmatic point of view, we believe that the individual faced with a diagnosis of pancreatic cancer would wish to be managed by a multidisciplinary team including a surgeon with specific training in this area and an annual caseload above a certain minimum threshold.

In conclusion, we have shown that surgically treated patients with pancreatic cancer are likely to fare better if they are managed by specialist pancreatic surgeons, or clinicians with an interest in this field. Although we cannot exclude entirely the possibility of bias or confounding, our results add to a growing body of evidence supporting specialisation of surgery for pancreatic malignancy. This has major implications for the delivery of cancer services in Scotland and the rest of the UK.
